# Comparison of organic acids supplementation on the growth performance, intestinal characteristics and morphology, and cecal microflora in broilers fed corn-soybean meal diet

**DOI:** 10.5713/ab.21.0448

**Published:** 2022-04-22

**Authors:** Hao Yang Sun, Hong Bin Zhou, Yang Liu, Yue Wang, Cheng Zhao, Liang Mei Xu

**Affiliations:** 1 Institute of Animal Nutrition, College of Animal Science and Technology, Northeast Agricultural University, Harbin 150030, China; 2 Dalian Chengsan Animal Husbandry Co., Ltd., Dalian 116308, China

**Keywords:** Cecal Microflora, Growth Performance, Intestinal Characteristics, Organic Acids

## Abstract

**Objective:**

The aim of this study was to compare the effects of three kinds of organic acid (OA) products on the growth performance, intestinal characteristics and morphology, and cecal microflora in broilers fed a corn–soybean meal meal diet.

**Methods:**

A total of 420 one-day-old male Cobb 500 broilers with an average initial body weight of 49.11±1.02 g were used in this 42-day experiment. Birds were randomly allotted to one of five treatments (7 replicates with 12 birds per replicate). Treatments consisted of negative control (NC), basal diet; positive control (PC), basal diet+100 mg/kg of Aviramycin; OA1, basal diet+500 mg/kg of OA product 1; OA2, basal diet+1,000 mg/kg of OA product 2; and OA3, basal diet+1,200 mg/kg of OA product 3.

**Results:**

The results indicated that OA product addition had no effect on growth performance parameters, such as body weight gain, feed intake, and feed conversion ratio, from days 1 to 14, 15 to 28, and 0 to 42, or on the pH values of the intestine, intestinal weight, or intestinal weight to body weight ratio. The intestinal morphology in terms of villus height and crypt depth were affected by dietary supplementation of OA products, respectively. Furthermore, dietary addition of OAs had positive influences on the maintenance of the cecal microflora based on the results of 16S rRNA analysis.

**Conclusion:**

Dietary inclusion of three kinds of OA products all benefit broilers, but the mode of action may be different. This study provides a basis for the application of OA products used in the poultry industry.

## INTRODUCTION

The short digestive tract of poultry leads to ineffective digestion and absorption, which is considered one of the key factors restricting the development of the modern poultry industry. Previously, dietary addition of subclinical doses of antibiotics was widely used to improve the performance and utilization in poultry; however, the overuse of antibiotics also leads to antibiotic resistance and residues in poultry intestines and products [1,2]. Organic acids (OAs) are organic compounds with acidic properties that are primarily composed of short-chain and medium-chain fatty acids [3]. During the last two decades, particularly after the ban of antibiotic growth promoter use in livestock animal feed, OAs have been widely used as feed additive to enhance the growth performance of poultry [4–7]. Researchers also found that dietary supplementation with OAs could enhance the nutrient digestion and absorption, improve feed intake (FI), and modulate the functions of the immune system [8–10]. Furthermore, it was reported that even when added to water, OAs also showed some positive results in affecting some designated beneficial intestinal bacteria and decreasing pathogenic bacteria [11].

During the past years, the application of different kinds of OAs has been reported to positively affect the performance, gastrointestinal development, immune system and antioxidant activity in broilers. For instance, Adil et al [12] reported that supplementation with butyric acid, fumaric acid and lactic acid all have beneficial effects on the growth performance of broilers. Mohammadagheri et al [13] determined that dietary inclusion of 10 g/kg citric acid decreased FI and increased villus height (VH) and width in broilers. Huang et al [14] indicated that dietary addition of benzoic acid improved the growth performance, jejunal enzyme activity and cecal *Escherichia coli* population. However, some other researchers have had conflicting results, such as Isabel and Santos, who reported that the blend of formic acid and propionic acid did not improve on growth performance in broilers [15]. Moreover, Houshmand et al [16] found that dietary addition of 1.5 g/kg of a balanced OA blend (formic acid, citric acid, malic acid, lactic acid, tartaric acid, and orthophosphoric acid) had no significant effects on performance, intestinal VH, crypt depth (CD), or gut pH in broilers.

Due to the huge market and types of OAs products, it is difficult for users to pick up the appropriate OA products. Therefore, to differentiate the effectiveness of different kinds of OA products; the aim of the current study was to compare effects of three common OA products in the market on the growth performance, intestinal characteristics and morphology, and cecal microflora in broilers fed a corn–soybean meal diet.

## MATERIALS AND METHODS

The experimental protocol used in this study was approved by the Animal Care and Use Committee of Northeast Agricultural University, People’s Republic of China (Approval Number: NEAU-(2018)-10). The experiment was carried out at A’cheng Experimental Base of Northeast Agricultural University.

### Experimental design, animals, diets, and housing

A total of 420 one-day-old male Cobb 500 broiler chickens with an average initial body weight (BW) of 49.11±1.02 g were used in a 42-day growth assay. Birds were randomly assigned to 1 of 5 dietary treatments (7 replicates and 12 broilers per replicates): NC, commercial basal diet; PC, NC+100 mg/kg of Avilamycin; OA1, NC+500 mg/kg of OA product 1; OA2, 1,000 mg/kg of OA product 2; OA3, 1,200 mg/kg of OA product 3. The experiment was conducted in three phases: phase 1 (days 1 to 14), phase 2 (days 15 to 28), and phase 3 (days 29 to 42). The base diets were corn-soybean meal diets. All diets were formulated to meet or exceed the National Research Council (1994) requirement for broiler chickens and were provided in mashed form. All the feed was fed daily and mixed daily. To ensure that the products could mixed well into the diets, the extract was first mixed with 1 kg feed by hand, and then this premix feed was mixed properly with the remaining feed using a mixer according to the manufacturer’s protocol [17]. The composition of the basal diet is shown in Table 1. Broilers were housed in a temperature-controlled room with 3 floors of stainless steel battery cages (1.75×1.55 m^2^). The temperature in the room was 33°C±1°C for the first 3 days and was then gradually decreased by 3°C per week to 20°C, which was maintained until the end of the experiment. The humidity was kept at approximately 60% throughout the experiment. The broilers had free access to feed and water during the experiment.

### Products information

The OA products used in this study were randomly chosen from each kind of product on the market. The OA1 product was a coating product with at least 350 g/kg of coated benzoic acid as active constituent. The OA2 product containeds active constituents of 280 g/kg formic acid and 350 g/kg calcium citrate. The chemical composition of the OA3 product was 100 g/kg of formic acid, 120 g/kg of citric acid, and 150 g/kg of lactic acid. The dosage of dietary supplementation in this experiment were followed the optimum supplemental levels in the product guidelines.

### Sampling and measurements

*Growth performance and intestinal characteristics*: On days 0, 14, 28, and 42, chickens were weighed by pen and FI was recorded to calculate body weight gain (BWG), FI, and feed conversion ratio (FCR). After 42 days feeding trial, two birds per replicate (14 birds per treatment) were randomly selected (n = 70) and then slaughtered by severing the jugular vein. The cecum and small intestine and their contents were sampled under aseptic conditions. The content samples were immediately transferred into liquid nitrogen and then stored at −80°C until further analysis. The intestine samples were fixed in 10% buffered formalin until analysis. The small intestine was separated into duodenum, jejunum, and ileum. After separation, each part of the small intestine and cecum was weighed, and the intestine to weight ratio (IWR) was then calculated. For the pH values, a glass-electrode pH meter (WTW pH 340-A; WTH Measurement Systems Inc., Ft. 165 Myers, FL, USA) was used, and the results were accurate to 0.01. For the intestinal morphology, at the middle position approximately 2 cm segments from the duodenum, jejunum, and ileum were collected. Samples were embedded in paraffin. A 6 μm section of each sample was placed onto a glass slide and stained with Alcian blue/hematoxylin and eosin for light microscope examination [18]. Villus height and CD of the samples were measured at 100× magnification using computer software (Motic Images Advanced 3.2; Motic, San Antonio, TX, USA), and then the villus height to crypt depth ration was calculated.

### Cecal microflora

Community DNA of the intestinal contents was extracted with a NucleoSpin 96 Plasmid Core Kit (case No, 740625.4; Macherey-Nagle CmbH & Co. KG, Germany), following the manufacturer’s instructions. Polymerase chain reaction (PCR) amplicons covering the hypervariable V3-V4 regions of the 16S rRNA gene from community DNA were amplified by using primer pairs 338F (5′-ACTCCTACGGGAGGCAGCA-3′) and 806R (5′-GGACTACHVGGGTWTCTAAT-3) modified to add adapters and unique barcodes for each sample. The PCR products were subsequently quantified, combined in equal amounts, and purified. FLASH v1.2.7 software was used to splice overlapping reads of each sample, and the splicing sequence obtained was the original tag (raw tag). To obtain clean reads, Trimmomatic v0.33 software was used to screen the raw tags. The low-quality reads and chimeric sequences were identified and removed by UCHIME v4.2 software. Based on the distance at 3# or less dissimilarity cutoff, sequences were clustered into operational taxonomic units (OTUs) by Uparse v7.0.1001. Then prokaryotic taxa were assigned to the representative sequence of each OUT using the SILVA database [19,20]. The taxa relative abundances of community composition in samples were identified at different levels respectively. Mothur software v1.30 was used to calculate the alpha diversity indices such as the Chaol, and Shannon indices and coverage rate.

### Statistical analysis

All data were subjected to statistical analysis in a randomized complete design using the mixed procedures [21]. The cage was used as the experimental unit for growth performance. For the cecal microflora and intestinal characteristics measurements, each individual bird was used as an experimental unit. Differences among treatment means were determined using Tukey’s test. Variability in the data were expressed as the pooled standard error of mean (SEM). p Values less than 0.05 were considered as statistically significant.

## RESULTS

As shown in Table 2, the results of growth performance indicated that from Days 1 to 14, 29 to 42, and 0 to 42, BWG, FI, and FCR were not affected by the treatments. From days 15 to 28, significant improvements were observed in the BWG of the PC treatment compared with these OA supplemented groups, whereas the FI and FCR were not influenced by any treatments.

**Figure 1:**
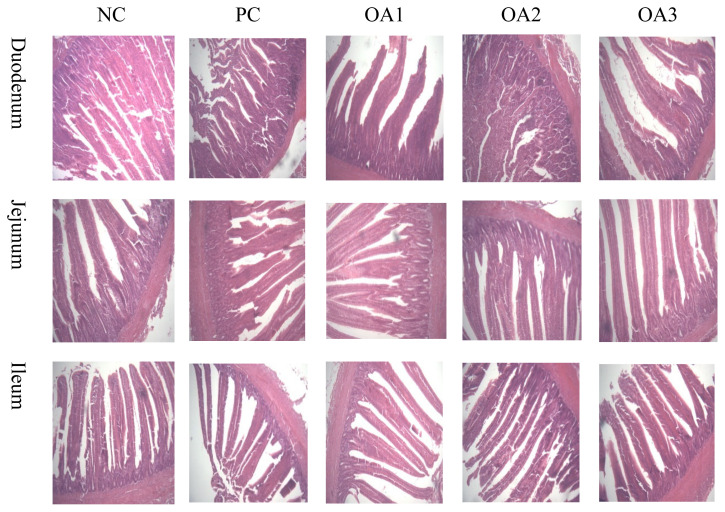
Photomicrographs of the comparison of organic acids on intestinal morphology of broilers. Sections were analyzed by optical microscopy at 100× for differences in intestinal morphology.

Tables 3 and 4 as well as Figure 1 show the intestinal characteristics of dietary OA supplementation in boilers. As presented in Table 3, OA3 group showed significantly (p<0.05) higher values in the duodenal VH and CD than the PC and OA1 groups; furthermore, the OA2 treatment also resulted in a higher (p<0.05) duodenal VH than the PC and OA1 treatments and higher (p<0.05) CD value than OA1 treatment. The jejunum VH values in the OA2 group were signidicantly (p<0.05) higher those in the NC group, whereas the CD and VH:CD values were not affected by any treatments. In the ileum, the OA2 and OA3 diets showed significantly (p<0.05) lower levels than the NC and PC diets. As described in Table 4, dietary inclusion of three kinds of OA products had no effects (p>0.05) on the pH values, weight, or IWR in broiler intestinal parameters.

**Figure 2:**
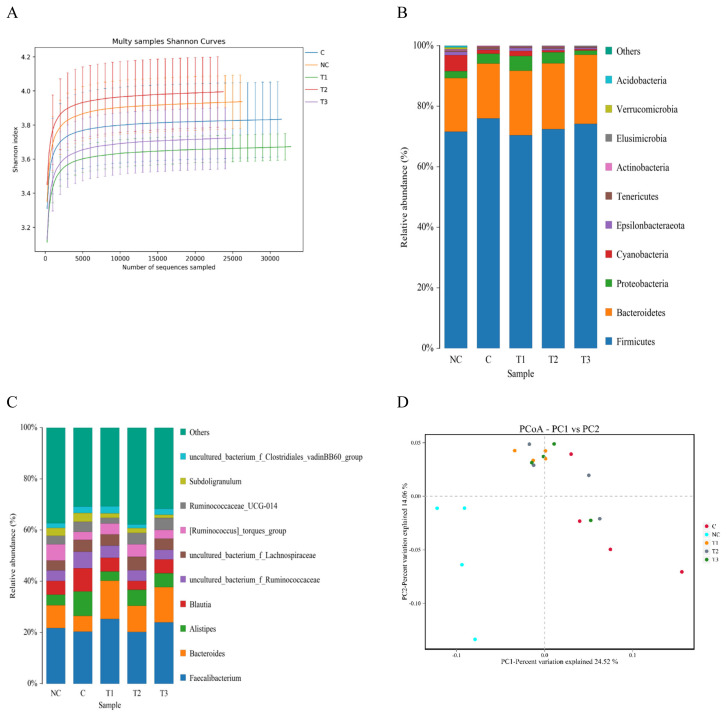
Shannon Index curves analysis of the cecum microflora of broilers fed five different diets (A); Community structure of phylum level (B); Community structure of genus levels (C); Principle coordinate analysis (PCoA), based on weighted UTP distances, of cecum microflora communities of broilers fed five different diets (D). At the phylum level, a higher percentage of Firmicutes was observed in the PC (75.82%), OA2 (72.56%), and OA3 (74.11%) groups than in the NC group (71.65%); moreover, the percentage of Bacteroidetes in the OA1 (21.19%), OA2 (21.58%), and OA3 (22.88%) groups were higher than those in the PC and NC. The community structure at the genus level were not affected by OA supplementation. NC, commercial basal diet; PC, NC+100 mg/kg of Avilamycin; OA1, NC+500 mg/kg of OA product 1; OA2, 1,000 mg/kg of OA product 2; OA3, 1,200 mg/kg of OA product 3.

**Figure 3:**
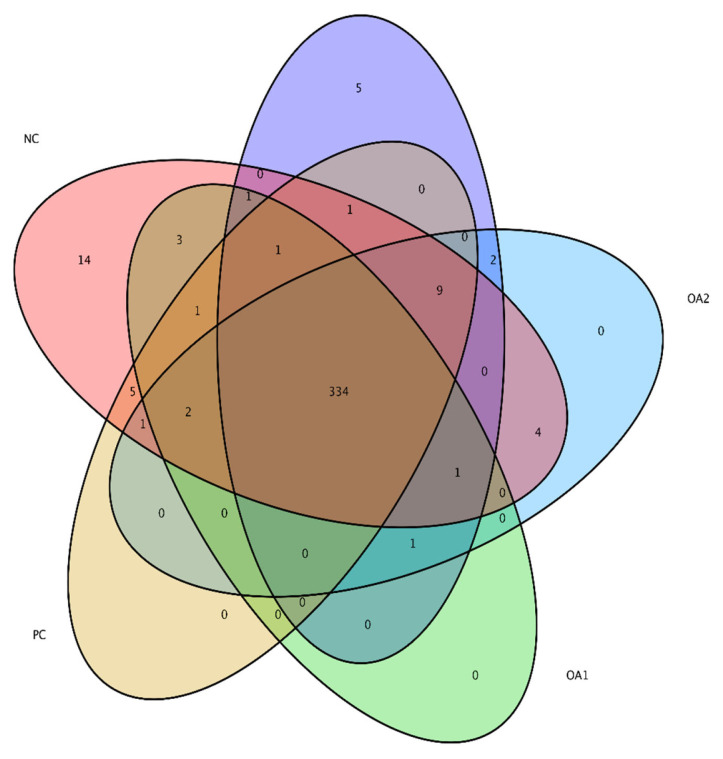
Venn diagram of alpha diversity of the microflora residing in the cecum of broilers at 42 days. The colors of the nodes indicate data types. Red indicating NC diets, yellow PC diets, green OA1 diets, blue OA2 diets, and purple OA3 diets. The community structure at the principle coordinate analysis were not affected by OA supplementation. NC, commercial basal diet; PC, NC+100 mg/kg of Avilamycin; OA1, NC+500 mg/kg of OA product 1; OA2, 1,000 mg/kg of OA product 2; OA3, 1,200 mg/kg of OA product 3.

The cecal microflora results are shown in Table 5, Figures 2 and 3. Regarding the alpha diversity, the Shannon and Simpson indices were not affected by any dietary treatment. The Chao1 and Ace indices in the PC, OA1, and OA2 groups were significantly (p<0.05) decreased compared with those in the NC group (Table 5). For the microflora community structure at the phylum level, a higher percentage of *Firmicutes* was observed in the PC (75.82%), OA2 (72.56%), and OA3 (74.11%) groups than in the NC group (71.65%); moreover, the percentage of *Bacteroidetes* in the OA1 (21.19%), OA2 (21.58%), and OA3 (22.88%) groups were higher than those in the PC and NC (Figure 2). However, the community structure at the genus level and the principle coordinate analysis were not affected by OA supplementation (Figures 2 and 3).

## DISCUSSION

The aim of the current study was to compare three kinds of OA products on the growth performance, intestinal characteristics and morphology, and cecal microflora in broilers fed a corn–soybean meal diet. Based on the growth performance results in this study, none of the three OA products had positive influences compared to no treatment and antibiotic treatment. In contrast to the results of the present study, many other studies observed significant improvements in growth performance with dietary supplementation with OAs. For instance, Gao et al [22] observed that dietary supplementation of 150, 200, and 250 mg/kg OA product (citric acid and sorbic acid) all showed improvement in BWG and FCR compared to control diet. Recently, Liu et al [9] indicated that dietary inclusion of 2 g/kg of lactic acid compound and formic acid both increased daily weight gain and decreased the ratio of daily FI to daily weight gain in broilers. Moreover, Ali et al [23] observed that dietary addition of 4 g/kg butyric acid glycerides not only improved the performance of normal birds but also enhanced the performance in broilers infected with *Eimeria maxima*. However, similar to the results of the present study Huang et al [14] also reported that dietary addition of two doses (7 g/kg and 14 g/kg) of benzoic acid did not affect the BWG and FRC in broilers during days 1 to 21. Recently, Lan et al [24] also suggested that dietary inclusion of three levels of sodium butyrate (0.3, 0.6, and 1.2 g/kg) had no effects on growth performance parameters including average daily gain, average daily feed intake, and FCR in broilers from days 22 to 45 and 1 to 45. The reasons for these inconsistent results may be attributable to the different feed ingredients and nutrient factors, composition of OAs and the mechanism of action of different OAs. It has been suggested that OAs in birds may decreases diet pH values and the antibiotic action of acids in birds [9]. The results of the present study may be due to the supplementation dosage of OAs being lower than that in other studies. Furthermore, the results of this study also indicate that the optimum supplemental levels stated in the OA product guidelines may serve as a safe dosage, but to achieve positive influences, higher level should be added according to the actual production conditions.

The intestine is one of the most important organs in animals for nutrient digestion and absorption. The growth and development of the intestine directly influences the nutrient metabolism and growth performance of broilers; therefore, integration into intestinal morphology and structure is the precondition to ensure broiler health [25]. In accordance with this experiment, Agboola et al [26] reported that dietary supplementation with a mixture of 40 g/kg formic acid and propionic acid improved the VH values of the ileum. Similarly, Ju et al [27] demonstrated that sodium butyrate addition significantly increased the VH values in the duodenum, jejunum and ileum, as well as the VH:CD in the jejunum and ileum in broilers. However, Giannenas et al [6] indicated that 3 g/kg of benzoic acid added to turkey diets did not affect the intestinal morphology. Moreover, Rodjan et al [28] showed that dietary inclusion of 2 g/kg OAs, including fumaric acid, formic acid, lactic acid, propionic acid and citric acid did not influence duodenal morphology in broilers. It has been suggested that a higher VH resulted in a higher capacity of enzyme secretion in enterocytes and better nutrient digestion and absorption [29]. On the other hand, CD values are considered an important indicator of the proliferation rate and maturity of enterocytes, and lower CD values indicate that the enterocyte proliferation rate is increased, which results in a stronger secretion function [30]. The differences among these studies may be due to the types, forms, and supplementation dosage of OAs, as well as the dietary formula and the interaction between diets and OAs.

The intestinal microflora is a complex ecosystem and is important for maintaining homeostasis of the gastrointestinal tract [31]. It also plays an important role in the health of the host by promoting the supply, digestion and absorption of nutrients, preventing pathogen colonization, and maintaining normal mucosal immunity [32]. It is widely accepted that OAs have antimicrobial action and are able to inhibit the growth of undesired pH-sensitive microorganisms; furthermore, it has been suggested that OAs also have a metabolic effect, serving as as energy source for the intestinal mucosa and modulating general metabolism [33]. In swine nutrition, many studies have investigated the effects of OA supplementation on gut microflora; however, few studies have illustrated the effects of OAs on intestinal microflora communities by sequencing-based techniques in broilers [11]. It was found that in the broiler cecum, *Bacteroidetes* and *Firmicutes* have the highest relative abundance [34]. The results of this experiment were in line with this finding. However, Oakley et al [35] demonstrated that both of OAs supplied as feed additive (propionic acid) and water supply (formic acid and propionic acid) had no effects on the cecal microflora in broilers over a 42-day experiment. Furthermore, it was also reported that the cecal microflora was drastically changed as a function of bird age [36]. The intestinal microflora is a complex ecosystem that varies among individuals and depends on host genotype and environmental factors, such as diet, antibiotics, and additives [37]. The changes in cecal microflora in this study may be relevant to the changes in intestinal morphology and the antimicrobial properties of OAs; however, further studies are still needed to determine the mechanism of action of OAs in the cecal microflora.

## CONCLUSION

In conclusion, dietary supplementation with these three kinds of OA products had no significant effects on growth performance or intestinal characteristics, such as intestinal weight, pH, and intestinal to BW ratio. The addition of these OAs has positive effects on the intestinal morphology and cecal microflora. Overall, dietary supplementation with OAs resulted in growth similar to that with antibiotics supplementation in broilers. Furthermore, the dosage of dietary supplementation is another key factor for the effects of OAs on broiler performance; based on the results of this experiment, the application of OAs in practice should be higher than the optimum supplemental levels in the product guidelines.

## Figures and Tables

**Table 1:** Ingredient composition of experimental diets (as-fed basis)

Items	Phase 1 (d 1 to 14)	Phase 2 (d 15 to 28)	Phase 3 (d 29 to 42)
Ingredients (%)			
Corn	61.77	62.31	63.14
Soybean meal	26.50	25.50	23.80
Corn gluten meal	4.70	1.50	3.00
Cottonseed meal	2.00	2.50	-
Limestone	0.50	0.80	0.80
Oil	2.50	5.20	7.00
Salt	0.25	0.25	0.25
DL-Methionine	0.56	0.64	0.68
L-Lysine	0.56	0.64	0.68
L-Threonine	0.16	0.16	0.15
Premix^1)^	0.50	0.50	0.50
Total	100.00	100.00	100.00
Calculated nutrient composition			
Metabolizable energy (kcal/kg)	2,975	3,100	3,150
Crude protein (%)	21.50	19.00	17.50
Calcium (%)	0.96	0.90	0.89
Lysine (%)	1.36	1.31	1.10
Threonine (%)	0.92	0.88	0.76
Methionine+cysteine	0.99	0.95	0.85

1) Provided per kg of complete diet: 12,000 IU vitamin A; 3,300 IU vitamin D_3_; 35 IU vitamin E; 1.2 mg vitamin K_3_; 7.2 mg riboflavin; 39 mg niacin; 0.06 mg biotin; 10.2 mg calcium pantothenic; 1.05 mg folic acid; 13 μg vitamin B12; 3.9 mg pyridoxine·HCL; 7.8 mg Cu; 65.5 mg Zn; 108 mg Mn; 1.05 mg I; 0.39 mg Se; 80 mg Fe.

**Table 2:** Comparison of organic acids supplementation on growth performance in 42-day-old broilers

Items	NC^1)^	PC^1)^	OA1^1)^	OA2^1)^	OA3^1)^	SEM	p-value
IBW (g)	48.98	49.06	49.40	48.94	49.18	1.04	-
Days 1 to 14							
BWG (g/bird)	399	396	400	395	390	8.00	0.922
FI (g/d/bird)	590	591	597	593	584	19.65	0.993
FCR	1.478	1.490	1.495	1.500	1.500	0.04	0.996
Days 15 to 28							
BWG (g/bird)	905^ab^	960^a^	870^bc^	932^bc^	814^c^	27.52	0.008
FI (g/bird)	1,348	1,436	1,304	1,381	1,196	62.44	0.127
FCR	1.489	1.498	1.500	1.543	1.475	0.05	0.909
Days 29 to 42							
BWG (g/bird)	1,136	1,148	1,176	1,098	1,190	52.85	0.760
FI (g/bird)	2,059	2,168	2,131	1,984	2,175	90.39	0.535
FCR	1.842	1.903	1.830	1.811	1.841	0.10	0.975
Day 0 to 42							
BWG (g/bird)	2,440	2,504	2,446	2,325	2,395	59.86	0.321
FI (g/bird)	3,997	4,195	4,032	3,858	3,956	121.5	0.414
FCR	1.641	1.678	1.650	1.661	1.651	0.04	0.979

The body weight gain expressed the average gain per bird.

SEM, pooled standard error of the mean; IBW, initial body weight; BWG, body weight gain; FI, feed intake; FCR, feed conversion ratio.

1) NC, commercial basal diet; PC, NC+100 mg/kg of Avilamycin; OA1, NC+500 mg/kg of OA product 1; OA2, 1,000 mg/kg of OA product 2; OA3, 1,200 mg/kg of OA product 3.

a–c Within a row, values not sharing a common superscript are significantly different at p<0.05.

**Table 3:** Comparison of organic acids supplementation on intestinal morphology in 42-day-old broilers

Items	NC^1)^	PC^1)^	OA1^1)^	OA2^1)^	OA3^1)^	SEM	p-value
Duodenum							
Villus height (μm)	3,431^ab^	3,268^b^	2,901^b^	4,045^a^	4,085^a^	239.4	0.008
Crypt depth (μm)	1,099^abc^	1,019^bc^	999^c^	1,322^ab^	1,389^a^	100.1	0.012
VH:CD	3.15	3.26	3.46	3.17	3.17	0.36	0.979
Jejunum							
Villus height (μm)	3,436^b^	3,730^ab^	3,699^ab^	4,336^a^	3,764^ab^	210.0	0.048
Crypt depth (μm)	1,253	1,100	1,101	1,157	1,013	116.7	0.686
VH:CD	3.02	3.58	3.50	3.98	3.81	0.38	0.479
Ileum							
Villus height (μm)	4,190^a^	3,964^a^	3,658^ab^	3,667^b^	3,190^b^	172.8	0.002
Crypt depth (μm)	906	690	735	659	883	84.8	0.171
VH:CD	4.88	6.62	5.25	5.57	3.62	0.76	0.127

SEM, pooled standard error of the mean; VH:CD, villus height to crypt depth ratio.

1) NC, commercial basal diet; PC, NC+100 mg/kg of Avilamycin; OA1, NC+500 mg/kg of OA product 1; OA2, 1,000 mg/kg of OA product 2; OA3, 1,200 mg/kg of OA product 3.

a–c Within a row, values not sharing a common superscript are significantly different at p<0.05.

**Table 4:** Comparison of organic acids supplementation on intestinal morphology in 42-day-old broilers

Items	NC^1)^	PC^1)^	OA1^1)^	OA2^1)^	OA3^1)^	SEM	p-value
Duodenum							
pH	5.39	5.65	5.55	5.50	5.60	0.101	0.457
Weight (g)	18.07	18.86	19.58	18.91	20.05	1.125	0.769
IWR (%)	0.79	0.71	0.77	0.81	0.80	0.001	0.698
Jejunum							
pH	5.49	5.48	5.37	5.37	5.36	0.055	0.287
Weight (g)	42.77	52.15	47.58	50.40	50.46	6.669	0.869
IWR (%)	1.85	1.94	1.88	2.15	2.01	0.003	0.931
Ileum							
pH	6.60	6.46	6.00	6.37	5.88	0.278	0.354
Weight (g)	29.30	39.23	33.37	38.04	37.55	0.326	0.256
IWR (%)	1.27	1.46	1.32	1.64	1.58	0.002	0.450
Cecum							
pH	6.41	6.05	6.00	6.37	5.88	0.295	0.650
Weight (g)	14.28	15.84	14.31	19.63	18.06	2.411	0.464
IWR (%)	0.63	0.58	0.57	0.82	0.72	0.001	0.295

SEM, pooled standard error of the mean; IWR, intestinal weight to body weight ratio.

1) NC, commercial basal diet; PC, NC+100 mg/kg of Avilamycin; OA1, NC+500 mg/kg of OA product 1; OA2, 1,000 mg/kg of OA product 2; OA3, 1,200 mg/kg of OA product 3.

**Table 5:** Comparison of organic acids supplementation on the Alpha diversity of cecum microflora in 42-day-old broilers

Item	NC^1)^	PC^1)^	OA1^1)^	OA2^1)^	OA3^1)^
Shannon	3.94±0.16	3.84±0.22	3.68±0.08	4.00±0.21	3.73±0.18
Simpson	0.07±0.02	0.06±0.02	0.09±0.02	0.06±0.02	0.09±0.02
Ace	350.36±3.72^a^	309.92±7.43^c^	326.34±6.58^bc^	325.03±7.49^bc^	332.68±4.78^ab^
Chao1	355.66±2.35^a^	320.02±6.54^c^	331.32±7.50^bc^	335.22±8.72^bc^	342.87±5.23^ab^

1) NC, commercial basal diet; PC, NC+100 mg/kg of Avilamycin; OA1, NC+500 mg/kg of OA product 1; OA2, 1,000 mg/kg of OA product 2; OA3, 1,200 mg/kg of OA product 3.

a–c Within a row, values not sharing a common superscript are significantly different at p<0.05.
